# Response-adaptive randomization in clinical trials: from myths to practical considerations

**DOI:** 10.1214/22-STS865

**Published:** 2023-05

**Authors:** David S. Robertson, Kim May Lee, Boryana C. López-Kolkovska, Sofía S. Villar

**Affiliations:** MRC Biostatistics Unit, University of Cambridge, Forvie Site, Robinson Way, Cambridge CB2 0SR, United Kingdom; MRC Biostatistics Unit; MRC Biostatistics Unit and is currently working at AstraZeneca; MRC Biostatistics Unit

**Keywords:** ethics, patient allocation, power, sample size imbalance, time trends, type I error control

## Abstract

Response-Adaptive Randomization (RAR) is part of a wider class of data-dependent sampling algorithms, for which clinical trials are typically used as a motivating application. In that context, patient allocation to treatments is determined by randomization probabilities that change based on the accrued response data in order to achieve experimental goals. RAR has received abundant theoretical attention from the biostatistical literature since the 1930’s and has been the subject of numerous debates. In the last decade, it has received renewed consideration from the applied and methodological communities, driven by well-known practical examples and its widespread use in machine learning. Papers on the subject present different views on its usefulness, and these are not easy to reconcile. This work aims to address this gap by providing a unified, broad and fresh review of methodological and practical issues to consider when debating the use of RAR in clinical trials.

## 1 Introduction

Randomization to allocate patients to treatments is a defining element of a well-conducted study, ensuring comparability of treatment groups, mitigating selection bias, and providing the basis for statistical inference (Rosenberger and Lachin, 2016). In clinical trials, a randomization scheme which remains unchanged with patient responses is still the most frequently used patient allocation procedure. Alternatively, randomization probabilities can be *adapted* during the trial based on the accrued responses, with the aim of achieving experimental objectives. Objectives that can be targeted with a Response-Adaptive Randomization (RAR) procedure include maximizing power of a specific treatment comparison and assigning more patients to an effective treatment during the trial.

Few topics in the biostatistical literature have received as much attention over the years as RAR (also known as outcome-adaptive randomization). RAR has been a fertile area of methodological research, as illustrated by the reference section of this paper. Despite this, the uptake of RAR in clinical trial practice remains disproportionately low in comparison with the theoretical interest it has generated since first proposed by Thompson (1933). Its value in clinical trials remains a subject of active debate within biostatistics, especially during health care crises such as the Ebola outbreak (Brittain and Proschan, 2016; Berry, 2016) or the COVID-19 pandemic (Proschan and Evans, 2020; Magaret, 2020; Villar et al., 2021).

This continued conversation has been enriching, but is also often presented in papers geared towards arguing either in favor or against its use in clinical trials, which has given RAR a controversial flavour. As well, some of these debates have been somewhat repetitive, as seen by how many of the points raised by Armitage (1985) over 35 years ago continue to be revisited. Examples of possibly conflicting views on the use of RAR are given below.

If you are planning a randomized comparative clinical trial and someone proposes that you use outcome adaptive randomization, Just Say No. (Thall, 2020)

…optimal [RAR] designs allow implementation of complex optimal allocations in multiple-objective clinical trials and provide valid tools to inference in the end of the trial. In many instances they prove superior over traditional balanced randomization designs in terms of both statistical efficiency and ethical criteria. (Rosenberger et al., 2012)

RAR is a noble attempt to increase the likelihood that patients receive better performing treatments, but it causes numerous problems that more than offset any potential benefits. We discourage the use of RAR in clinical trials. (Proschan and Evans, 2020)

The above examples help explain why the use of RAR in clinical trials remains rare and debated. It also suggests that, given the many different classes of RAR that exist, making general statements around the relative merits of RAR may well be an elusive goal. This paper therefore aims to give a balanced and fresh perspective. Instead of conveying a position in favor or against the use of RAR in clinical trials in general, we emphasize the less commonly known arguments (which also tend to be ones that are more positive towards the use of RAR).

In parallel and in stark contrast to this discussion, in machine learning the uptake and popularity of Bayesian RAR (BRAR), also referred to as Thompson Sampling (TS), has been incredibly high (Kaufmann and Garivier, 2017; Kaibel and Biemann, 2021; Lattimore and Szepesvári, 2020). Their use in practice has been driven by substantial gains in system performances. Meanwhile, in the clinical trial community, a crucial development was the use of BRAR in some well-known biomarker led trials such as I-SPY 2 (Barker et al., 2009) or BATTLE (Kim et al., 2011). The goal of these trials was to learn which subgroups (if any) benefit from a therapy and to change the randomization to favor patient allocation in that direction. While these trials include other elements besides RAR, they have set a precedent that RAR is feasible (at least in oncology), and have set expectations which, contrary to what the ECMO trials did in the 1980s (see Section 2), are driving investigators towards RAR in other contexts. Both in the machine learning literature and in these trials, the BRAR methodology used is a subclass of the larger family of RAR methods.

After an extensive review of the literature, we recognized the need for an updated and broad discussion aimed at reconciling apparently conflicting arguments. We believe this is important because some of these (mostly negative) positions on RAR persist, despite recent methodological developments over the past 10 years directly addressing past criticisms (see for example Section 3.4). We compare recently proposed RAR procedures and use a new simulation study (in Section 3.1) to illustrate how some viewpoints can tell only part of the story while a broad look can change conclusions. Additionally, we hope this paper will drive methodological research towards areas that are less developed and help those considering the use of RAR in a specific experiment to navigate the relevant literature in light of recent opposing views (Proschan and Evans, 2020; Villar et al., 2021; Magaret, 2020). Overall, our ultimate message is a call for careful thinking about how to best deliver experimental goals through the appropriate use of trial adaptations including (but not limited to) RAR.

We end this section by providing some general notation, basic concepts and metrics to assess RAR. We give a historical overview of RAR in Section 2, including a summary of classification criteria of different procedures (Section 2.3). We subsequently explore some key established views about RAR in Section 3. We conclude with final considerations and a discussion in Section 4.

### 1.1 Some notation and basic concepts

We first describe the setting and notation necessary for a rigorous presentation of the debate around RAR. Note that Table A1 in the Appendix provides a summary of all the acronyms used in this paper. Our focus is on clinical trials in which a fixed number of experimental treatments (labeled 1, …, *K* with *K* ≥ 1) are compared against a control or standard of care treatment (labeled 0) in a sample of *n* patients. The sample size *n* is also assumed fixed. This can, in principle, be relaxed to allow for early stopping of the trial, but for the purposes of this paper we consider early stopping as a distinct type of adaptation. When treatment *k* ∈ {0, 1, …, *K*} is assigned to patient *i* (for *i* ∈ {1, …, *n*}), this generates a random response variable *Y_k,i_*, which represents the primary outcome measure of the clinical trial.

We let *a_k,i_* be a binary indicator variable denoting the observed treatment allocation for patient *i*, with *a_k,i_* = 1 if patient *i* is allocated to treatment *k* and *a_k,i_* = 0 otherwise. Each patient is allocated to one treatment only, and hence $\sum_{k=0}^{K}a_{k,i}=1$. Typically patients enter the trial and are treated sequentially, either individually or in groups. In most of the RAR literature, patients are assumed to be randomized and treated one after another, with each patient’s outcome being available before the next patient needs to be treated. This assumption can be relaxed and incorporate delayed patient outcomes (e.g. for time-to-event data).

We assume *Y_k,i_* depends on a treatment-specific parameter of interest *θ_k_*. For notational convenience, we let *Y_i_* denote the realised outcome of patient *i*. We assume a parametric model for the primary outcome, ignoring nuisance parameters and other parameters of secondary interest for the final analysis. For example, one could have a Bernoulli model for binary responses, where *θ_k_* = *p_k_* (the probability of a successful outcome for a patient on treatment *k*):

(1) $$\begin{array}{ll} Pr\left(Y_{k,i}=y|a_{k,i}=1\right)=p_{k}^{y}{\left(1-p_{k}\right)}^{\left(1-y\right)} & \text{for}y=0,1. \end{array}$$

Other examples include a normal or exponential model for continuous outcome variables.

As a general way to represent treatment allocation rules, we let *π_k,i_* = *P* (*a_k,i_* = 1) denote the probability that patient *i* is allocated treatment *k*. Note that we require $\sum_{k=0}^{K}\pi_{k,i}=1$ and *π_k,i_* > 0 ∀*i*. Also note that our definition excludes non-randomized response-adaptive methods like the Gittins Index (Villar et al., 2015a). Traditional (fixed) randomization has *π_k,i_* = *c_k_* for all *i*, and for implementing Equal Randomization (ER) we set *c_k_* = 1/(*K* + 1) for all *k*. Finally, we let *N_k_* denote the total number of patients that are allocated to treatment *k* by the end of the trial. In general, $N_{k}=\sum_{i=0}^{n}a_{k,i}$ is a random variable, with the constraint $\sum_{k=0}^{K}N_{k}=n$.

In a RAR procedure, the allocation probabilities that define the *sampling strategy* are adapted based on the past treatment allocations and response data. More formally, let ***a_i_*** = (*a*_0,*i*_, *a*_1,*i*_, …, *a_K,i_*) denote the allocation vector for patient *i*. We also let ***a***^(*j*)^ = {***a*_1_***, …, **a_j_***} and ***y***^(*j*)^ = {*y*_1_, …, *y_j_*} denote the sequence of allocations and responses observed for the first *j* patients (where both ***a***^(0)^ and ***y***^(0)^ are defined as the empty set). RAR defines the allocation probability *π_k,i_* conditional on ***a***^(*i*−1)^ and ***y***^(*i*−1)^, i.e.

(2) $$\pi_{k,i}=Pr\left(a_{k,i}=1|a^{\left(i-1\right)},y^{\left(i-1\right)}\right).$$

Note that for a procedure to be response-adaptive, the *π_k,i_* must depend on both ***a***^(*i*−1)^ and ***y***^(*i*−1)^. This framework is flexible enough to allow for the RAR procedure to also depend on covariates that may affect the primary outcome. Letting ***x***^(*j*)^ = {***x***_1_*, …, **x**_j_*} be a vector of observed covariates, we define a Covariate-Adjusted Response-Adaptive (CARA) procedure by letting *π_k,i_* = *Pr*(*a_k,i_* = 1 | ***a***^(*i*−1)^, ***y***^(*i*−1)^*, **x***^(*i*)^). With the increasing interest in “precision medicine”, the role of covariates is crucial in developing targeted therapies for patient subgroups. Many of the issues we discuss here for RAR are directly applicable (to some degree) to CARA. However, we do not include a specific discussion for CARA to preserve the focus of our work on RAR. We instead refer the reader to the review by Rosenberger and Sverdlov (2008), more recent papers by Atkinson, Biswas and Pronzato (2011); Baldi-Antognini and Zagoraiou (2011, 2012); Metelkina and Pronzato (2017) and the book by Sverdlov (2016). Zagoraiou (2017) discusses how to choose a CARA procedure in practice.

A final concept to introduce is that of hypothesis testing. We focus on the case where there is a global null hypothesis 𝓗_0_: *θ_k_* = *θ*_0_ ∀*k* versus one-sided alternatives 𝓗_1,*k*_: *θ_k_* > *θ*_0_ for some *k* (assuming a larger value of *θ_k_* represents a desirable outcome). At the end of the trial, a test statistic denoted *T_n_* = *t*(***a***^(*n*)^, ***y***^(*n*)^) is computed based on the observed data. The specific form of the test statistic depends on the outcome model and the hypothesis of interest. For example, if the primary outcome is binary, the Maximum Likelihood Estimator (MLE) of the success rate on treatment *k* is ${\hat{p}}_{k}=\frac{\sum_{i=1}^{n}a_{i,k}y_{i,k}}{\sum_{i=1}^{n}a_{i,k}}$. In a two-arm trial, one could use a *Z*-test based on the MLE of the success rates:

(3) $$Z_{n}=\frac{{\hat{p}}_{1}-{\hat{p}}_{0}}{\sqrt{{\hat{p}}_{0}\left(1-{\hat{p}}_{0}\right)/N_{0}+{\hat{p}}_{1}\left(1-{\hat{p}}_{1}\right)/N_{1}}}.$$.

### 1.2 Assessing the performance of RAR procedures

In the literature, many ways of assessing RAR have been considered. Most metrics used in the clinical trial setting focus on inferential goals. Terms such as ‘power’ and ‘patient benefit’ can have very different meanings depending on the trial context. Here, rather than providing an exhaustive list of all possible metrics for comparing variants of RAR, we present some of the most relevant ones in three categories: *testing*, *estimation* and *patient benefit*.

#### Testing metrics: type I error and power

For confirmatory trials, the control of frequentist errors is especially important from a regulatory perspective. A type I error is defined as falsely rejecting a null hypothesis 𝓗_0_. For a trial with a single null hypothesis 𝓗_0_: *θ* = *θ*_0_, the type I error rate is defined as *α* = *Pr*(rejecting 𝓗_0_ | *θ* = *θ*_0_), and for confirmatory trials this is controlled below some fixed level (typically 0.05 or 0.025). When there are multiple null hypotheses, various generalizations can be considered, the most common being the familywise error rate, which is the probability of making at least one type I error. This reflects the inherent multiplicity problem and type I error inflation that can occur if multiple hypotheses are tested without adjustment.

In contrast, a type II error is failing to reject 𝓗_0_ when it is in fact false. For a trial with a single null hypothesis 𝓗_0_ and corresponding point alternative hypothesis 𝓗_1_: *θ* = *θ*_1_, the power of the trial is defined as 1 − *β* = *Pr*(rejecting 𝓗_0_ | *θ* = *θ*_1_). However, when there are multiple hypotheses (e.g. in the multi-arm setting with *K* > 1), the ‘power’ of the trial admits various definitions. For instance, marginal power (the probability of rejecting a particular non-null hypothesis), disjunctive power (the probability of rejecting at least one non-null hypothesis) and conjunctive power (the probability of rejecting all non-null hypotheses) are all used as definitions of ‘power’ (Vickerstaff et al., 2019). Additionally, some authors define power as the probability of satisfying a criterion that reflects the goal of the trial. For example, power could be defined as the probability of selecting the best experimental treatment at the end of the trial, or as a Bayesian concept such as posterior predictive power. A RAR procedure can have a high power according to one definition but not according to another.

#### Estimation metrics

There are metrics related to estimation and the information gained after a trial. A key consideration (particularly for adaptive designs, see Robertson et al. (2021)) is bias, defined as a systematic tendency for the estimate of the treatment effect to deviate from its true value. More formally, the mean bias of an estimator ${\hat{\theta }}_{k}$ for *θ_k_* is defined as $E\left({\hat{\theta }}_{k}\right)-\theta_{k}$. An estimator may be biased due to the trial adaptations affecting its sampling distribution, or due to heterogeneity in the observed data (i.e. where the data does not come from the same underlying distribution, such as when there is a time trend in the response variable as considered in Section 3.4). Apart from bias, another important consideration is the variance $\mathrm{var}\left({\hat{\theta }}_{k}\right)$ or mean squared error of an estimator $E\left[\left({\hat{\theta }}_{k}-\theta_{k}{}^{2}\right)\right]$, reflecting the classical bias-variance trade-off. Although precision of the estimates is less often reported in the literature, this can be compared using estimation efficiency measures, see for example Flournoy et al. (2013); Sverdlov and Rosenberger (2013a).

#### Patient benefit metrics

Different metrics to capture the “ethical” or patient benefit properties of RAR have been considered. These are less frequently reported than testing and estimation metrics, which is somewhat counter-intuitive given the most common motivation to use RAR is to better treat more patients in a trial. Nevertheless, this lack of reporting is consistent with inferential goals being paramount. Some examples of patient benefit metrics include:

- The number of treatment successes (for binary outcomes) or the total response (for continuous outcomes) in the trial: $\sum_{i=1}^{n}Y_{i}$. When averaged for binary outcomes, this is referred to as the Expected Number of Successes (ENS). Alternatively, some authors focus on the number of treatment failures $\sum_{i=1}^{n}\left(1-Y_{i}\right)$ and report the Expected Number of Failures (ENF).
- The proportion of patients allocated to the best arm: $p^{*}=\sum_{i=1}^{n}a_{i,k^{*}}/n$, where k* = argmax*_k_θ_k_* (if k* is not unique then one option is to sum over all arms that are ‘best’).

The above metrics are concerned with the individual ethics of the *n* patients within the trial, which is distinct from the collective ethics of the overall population (which is related to testing and estimation metrics). We return to this issue of patient horizon in Section 3.6.

#### Other metrics

Aside from the three categories of metrics described above, there are also metrics focusing on the level of imbalance in the number of patients in each treatment arm at the end of the trial. One way of defining the imbalance in arm *k* is (*N_k_/n* − 1/(*K* + 1)), which makes a comparison between the observed allocation ratio and a completely balanced allocation between the arms. See also Section 3.1 for other examples of imbalance metrics.

A final metric is the total *sample size* of the trial. Typically, this is defined as the minimum number of patients required to achieve a target power (given type I error constraints) under some pre-specified point alternative hypothesis. This is closely linked with testing metrics but there are patient benefit considerations as well. For example, suppose one out of the (*K* + 1) treatment options is substantially better than the rest. Using ER means that *Kn/*(*K* + 1) of the patients within the trial will be allocated to suboptimal treatments. Hence, minimizing the sample size *n* has patient benefit advantages as well. In contrast (as discussed in (Berry and Eick, 1995)), increasing the sample size to maintain power when using RAR may deliver higher overall patient benefit across the target population (i.e. including future patients), suggesting trade-offs between benefit for patients in the trial and those outside of it, see also Section 3.6.

## 2 A Historical Perspective on RAR

“Those who cannot learn from history are doomed to repeat it.” (Attributed to George Santayana)

**FIG 1:**
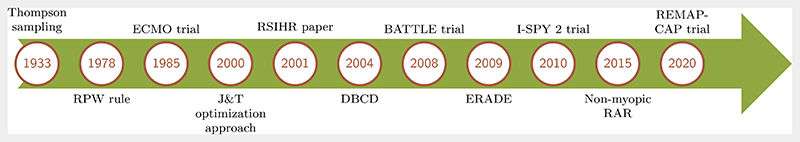
Timeline summarizing some of the key developments around the theory and practice of RAR in clinical trials. J&T = Jennison and Turnbull (2000), RSIHR = Rosenberger et al. (2001a).

We now give an overview of the historical development of RAR, which naturally motivates how we classify RAR procedures in Section 2.3. A distinguishing feature of this history is that a large amount of high quality theoretical work is paired with few highly influential examples of RAR in practice. We thus present the history of RAR in two distinct areas: theory (Section 2.1) and practice (Section 2.2). A timeline summarizing some key developments is given in Figure 1.

### 2.1 RAR methodology

The origins of RAR date back to Thompson (1933), who suggested allocating patients to the more promising treatment arm via a posterior probability computed using interim data. RAR seems to have been the first form of an *adaptive design* ever proposed. Another influential early procedure was the play-the-winner rule, proposed by Robbins (1952) and then Zelen (1969). Although partially motivated by Thompson’s idea, this is a non-randomized (deterministic) rule, where a success on one treatment leads to the subsequent patient being assigned to that treatment, while a failure leads to the subsequent patient being assigned to the other treatment.

RAR also has roots in the methodology for sequential stopping problems (where the sample size is random), as well as bandit problems (where resources are allocated to maximize the expected reward). Since most of the earlier work in these areas is non-randomized (i.e. concerns deterministic solutions), we do not review them here. Rosenberger and Lachin (2016, Section 10.2) gives a brief summary of the history of both of these areas, and an overview of multi-arm bandit models is presented in Villar et al. (2015a). For a review of non-randomized algorithms for the two-arm bandit problem, see Jacko (2019).

An important development for the clinical trials setting was the introduction of randomization to otherwise deterministic response-adaptive procedures. Randomization is essential for mitigating biases and ensuring comparability of treatment groups and is the default patient allocation mode in confirmatory clinical trials (Rosenberger and Lachin, 2016). An example of this is the Randomized Play-the-Winner (RPW) rule proposed by Wei and Durham (1978). The RPW rule can be viewed as an *urn model*: each treatment allocation is made by drawing a ball from an urn (with replacement) and the composition of the urn is updated based on the responses. In the following decades, many RAR rules based on urn models were proposed, with a focus on generalizing the RPW rule. We refer to Hu and Rosenberger (2006, Chapter 4) and Rosenberger and Lachin (2016, Section 10.5) for a detailed description.

Urn-based RAR procedures are intuitive, but are not optimal designs in a formal mathematical sense (see Section 2.3). From the early 2000s a perspective on RAR emerged based on *optimal allocation targets*, which are derived as a solution to a formal optimization problem. For two-arm group sequential trials, a general optimization approach was proposed by Jennison and Turnbull (2000, 2001), which minimizes the expected value of a loss function which is an arbitrary weighted average of *N*_0_ and *N*_1_. This led to the development of a whole class of optimal RAR designs. An early example for two-arm trials with binary outcomes is Rosenberger et al. (2001a). More examples are given in Section 3.2. In order to implement optimal allocation targets, a key development was the modification by Hu and Zhang (2004a) of the Doubly-adaptive Biased Coin Design (DBCD) originally described by Eisele (1994). Subsequent theoretical work by Hu and Rosenberger (2006) focused on asymptotically best RAR procedures, i.e. those with minimum asymptotic variance of the optimal allocation ratio (which typically depends on unknown parameters that need to be estimated using the response data, see the equations in Section 3.2). This led to the development of the class of efficient RAR designs (known as ERADE) proposed by Hu et al. (2009).

All the RAR procedures above are *myopic*, in that they use past responses *Y_k,i_* and past allocations *a_k,i_* to determine the allocation probabilities *π_k,i_*, without considering future patients to be recruited into the trial and the information they could provide. A recent development is non-myopic or *forward-looking* RAR based on solutions to the multi-bandit problem. The first such procedure was by Villar et al. (2015b) for binary responses, with subsequent work by Williamson et al. (2017) accounting for a finite time-horizon and for normally-distributed outcomes (see Williamson and Villar (2020)).

### 2.2 RAR in clinical practice

One of the earliest uses of RAR in clinical practice was the ECMO trial (Bartlett et al., 1985). This trial used the RPW rule on a study of critically ill babies randomized either to ECMO or to the conventional treatment. In total, 12 patients were observed: one in the control group, who died, and 11 in the ECMO group, who all survived. This extreme imbalance in sample sizes was a motivation for running a second randomized ECMO trial, using fixed randomization (Ware, 1989).

These ECMO trials have been the focus of much debate, with these two papers accruing over 1000 citations. Indeed, to this day the first ECMO trial is regarded as a key reason not to use RAR in clinical practice, due to the extreme treatment imbalance and highly controversial interpretation (Burton et al., 1997). Most recently, Proschan and Evans (2020) states “[RAR] had an inauspicious debut in the aforementioned ECMO trial”. Largely due to the controversy around these trials, there was little use of RAR in clinical trials for the subsequent 20 years. The pace of methodological work on RAR and adaptive designs more generally was negatively impacted as well (Rosenberger, 2015). One exception was the Fluoxetine trial (Tamura et al., 1994), which again used the RPW rule, but with a burn-in period to avoid large imbalances in treatment groups. For an in-depth discussion of both trials we refer to Grieve (2017), which also discusses two BRAR trials from the early 2000s.

More recently, there have been high-profile clinical trials that use BRAR as a key (but not the only) part of their adaptive design. Some examples in oncology include the BATTLE trials and the I-SPY 2 trial. The BATTLE trials (Kim et al., 2011; Papadimitrakopoulou, 2016) used RAR based on a Bayesian hierarchical model, where the randomization probabilities are proportional to the observed efficacy based on the individual biomarker profiles. Similarly, the I-SPY 2 trial (Barker et al., 2009) used RAR based on Bayesian posterior probabilities specific to different biomarker signatures. These trials have generated valuable discussions about the benefits and drawbacks of using RAR in clinical trials (Das and Lo, 2017; Korn and Freidlin, 2017; Siu et al., 2017). Meanwhile, the REMAP-CAP platform trial (Angus et al., 2020) also incorporated BRAR as part of its design, in the context of community-acquired pneumonia. This trial was subsequently tailored to respond to the COVID-19 pandemic (REMAP-CAP Investigators, 2021).

Although the BATTLE, I-SPY 2 and REMAP-CAP trials use RAR as part of their designs, their primary focus was to select optimal treatments for particular biomarker signatures, and hence can more precisely be described as master protocol trials (Woodcock and LaVange, 2017). Arguably the main feature of I-SPY 2 was the mechanism to ‘graduate’ or drop treatments and to add new ones as they arise. For recent examples of clinical trials using BRAR in a ‘vanilla’ fashion (although still including early stopping rules), we refer to Faseru et al. (2017); O’Brien et al. (2019); Barohn et al. (2021).

### 2.3 Classifying procedures: a taxonomy of RAR

Some papers (perhaps unintentionally) criticize the use of RAR in general or make broad conclusions using arguments that only apply to a specific class of procedures, as is (still) the case for the RPW rule and the ECMO trial (Proschan and Evans, 2020). In reality, RPW is just one example of a RAR procedure out of many and hence the value of other RAR procedures that are markedly different is harder to see. The vast number of different RAR procedures is a challenge that non-experts and experts alike face with when exploring the literature, which has accumulated (and continues to quickly evolve).

We now define several families of RAR procedures and discuss how they fit different classification criteria. This discussion illustrates the wealth and breadth of RAR methodology and its importance when assessing its value for a specific application. However, the criteria are not exhaustive or able to completely differentiate all types of RAR. As discussed next, we expect most classifications to require frequent revisiting given the current pace of development in the area (Villar et al., 2021). Nevertheless, these classifications can allow a better understanding of the many existing approaches and how they compare. We note that the number of references of each RAR family throughout the paper is a reflection of the attention each method received in the past rather than an intended focus.

#### Optimal and design-driven RAR

An important broad distinction first described by Rosenberger and Lachin (2002); Hu and Zhang (2004a) is between ‘optimal’ and ‘design-driven’ RAR. In their works, this is defined as the following.

1. ‘*optimal*’ RAR is based on deriving an optimal allocation target (or a sampling ratio), by optimizing a specific criterion based on a population response model.
  E.g. In Rosenberger et al. (2001a) an optimal RAR is defined for a two-arm trial based on the population model for binary responses. The power at the end of the trial (using a *Z*-test as given in equation (3)) is fixed, while the ENF is minimized. Formally, using the notation in Section 1.1 and defining *ρ* = *N*_1_/*n*, the optimization problem is as follows:
  (4) $$\begin{array}{lll} \underset{\rho }{\min}\left\{\left(1-p_{0}\right)N_{0}+\left(1-p_{1}\right)N_{1}\right\} & \text{subject}\mathrm{to} & \frac{p_{0}\left(1-p_{0}\right)}{N_{0}}+\frac{p_{1}\left(1-p_{1}\right)}{N_{1}}=C \end{array}$$
  The solution ρ* is then the optimal target ratio (given the above optimization criteria). To implement this in practice, it is necessary to estimate the parameters *p*_0_ and *p*_1_.
2. *‘design-driven’* RAR is based on rules which are established with intuitive motivation, but are not optimal in a formal sense.
  E.g. The RPW rule for binary responses. The rules for computing and choosing the allocation probability can be formulated using an intuitive urn-based model (see Section 2.1 and Wei and Durham (1978) for details).

A key difference for these two RAR classes is the computation of allocation probabilities. While approaches in family (1) rely on optimizing some objective function that describes aspects of the population model explicitly, those belonging to family (2) typically have an intuitive motivation that is not defined analytically from a population model. However, while classifying approaches into these two families is useful, there are some important caveats. First, an intuitive design may eventually be formally shown optimal in some sense. Second, some procedures are harder to classify into the above criteria. Consider bandit-based designs, such as the Forward-Looking Gittins Index (FLGI) rule in (Villar et al., 2015b) or the design by Williamson et al. (2017). These are based on an optimization approach but do not explicitly target a pre-specified optimal allocation ratio like in family (1). In certain cases (like for FLGI), these are heuristic approximations and can be viewed as having a more intuitive motivation.

An final caveat is that there are different optimality notions to consider. *Asymptotic* optimality for example was first introduced by Robbins (1952). For example, TS is asymptotically optimal in terms of minimizing cumulative regret (see e.g. Kaufmann et al. (2012)). So for large trials, one could consider it as belonging to family (1). However, in small samples, if TS (and its generalization proposed by Thall and Wathen (2007)) is used for assigning more patients to the better arm, then this would be closer an intuitive motivation (as in family 2), as only dynamic programming achieves ENS optimality in a finite sample.

#### Parametric and non parametric RAR

A classification that follows naturally from the previous one is that of *parametric* and *non-parametric* (or *distribution free*) RAR. This classification captures some of the spirit of the *optimal* versus *design-driven* while being possibly less subject to caveats. Parametric RAR procedures rely on assumptions that the response data are drawn from a given parametric probability distribution to compute and update the allocation probabilities *π_k,i_*.

E.g. the optimal RAR procedure proposed in Rosenberger et al. (2001a) and defined above requires estimates of *p*_0_ and *p*_1_ in order to determine *π_k,i_*.

In contrast, non-parametric RAR procedures do not explicitly rely on a parametric probability distribution nor on the corresponding parameter estimates to compute and update *π_k,i_*.

E.g. the RPW rule (and urn designs) are non-parametric designs that can be used for any binary data, regardless of the underlying probability distribution.

#### Bayesian and frequentist RAR

The distinction of RAR based on the frequentist or Bayesian approach to statistics may apply to the inference procedure used for the final analysis and/or to the design of the RAR itself. In our opinion, the inferential classification may not be helpful, since the choice of inference procedure depends on the experimental goals and regulators’ preferences between these approaches. Moreover, some innovative approaches have Bayesian design aspects but the inference focuses on the frequentist operating characteristics, see e.g. Ventz et al. (2017). Arguably a more relevant element to consider is the objective(s) of RAR (see the subsection ‘RAR with different objectives’ below). Readers interested in understanding the pros and cons of frequentist and Bayesian inference are referred to Wagenmakers et al. (2008); Samaniego (2010) as this is outside the scope of our review. For references on the use of Bayesian designs in the clinical trial context, we refer to Chow and Chang (2007); Chevret (2012); Rosner (2020); Stallard et al. (2020).

A common definition of a Bayesian design is that a prior distribution is explicitly incorporated into the design criteria/optimization problem and/or into the calculation of the allocation probabilities. However, the use of a prior distribution is not the defining element of BRAR as one can sometimes find equivalent frequentist designs using penalized MLEs or a specific prior distribution. For example, where the posterior mode with a uniform prior coincides with the MLE in a RAR procedure then an update of probabilities is the same from a frequentist and Bayesian perspective (see also a hybrid formulation for the RPW rule given in Atkinson and Biswas (2014, pg. 271)).

Hence, in the context of RAR, we define a Bayesian design as “a design rule that depends recursively on the posterior probability of the parameters” (Atkinson and Biswas, 2014), where the recursive updating of the allocation probabilities is done via Bayes Theorem. The prior information itself can be updated at time points when accrued trial data is available, see Sabo (2014). Such designs are called “fully Bayesian” in Ryan et al. (2016), and allow the full probabilistic description of all uncertainties, including future outcomes (i.e. predictive probabilities).

E.g. In TS with *K* = 1 and binary responses, the randomization probability is the posterior probability that *p*_1_ > *p*_0_ (given the prior information and available trial data), i.e. *π*_1,*i*_ = *P*(*p*_1_ > *p*_0_ | ***a***^*i*−1^, ***y***^*i*−1^).

A RAR procedure is *frequentist* if a frequentist approach is used for both estimating the unknown parameter(s) and, more importantly, for updating the allocation probabilities.

E.g. the DBCD can be used to target different allocations (see Section 3.2), where the *π_k,i_* are given as functions of the MLE.

#### RAR methodological families

RAR procedures can be classified in terms of the broad methodological ‘families’ they belong to: RAR based on TS (e.g. those suggested by Thall and Wathen (2007)), RAR based on urn models (e.g. RPW), RAR that target a pre-specified (optimal) allocation ratio (e.g. as in Hu and Zhang (2004a)) or bandit-based RAR procedures (e.g., the FLGI). This classification naturally follows from the historical developments in the area. However, RAR procedures could conceivably belong to more than one family and new types of RAR are continuously being developed.

#### RAR with different objectives

RAR procedures differ in the goal they are designed to achieve, either formally or intuitively. While some consider competing objectives such as both power and patient benefit (see Section 1.2 for definitions), others prioritize one over the other. Additionally, procedures can be non-myopic or myopic in their objective formulation. For some RAR procedures, such as those targeting an optimal allocation, the optimization problem can account for multiple objectives, see e.g. Hu et al. (2015); Baldi-Antognini and Giovagnoli (2010, 2015). More generally, within a Bayesian framework there is scope for composite utilities for multi-objective experiments (McGree et al., 2012; Baldi-Antognini and Giovagnoli, 2015; Metelkina and Pronzato, 2017). The selection of an objective may also require computational considerations.

Therefore, a good classification for comparing performance of RAR procedures is that of *single* objective procedures versus those that have *composite* objectives (reflecting trade-offs and constraints between possibly competing goals of an experiment).

E.g. FLGI in Villar et al. (2015b) has a non-myopic patient benefit goal, while Neyman allocation (see Section 3.2) has a power goal.

E.g. the design in Williamson et al. (2017) has a non-myopic patient benefit goal subject to a power constraint, while the ‘optimal’ allocation of Rosenberger et al. (2001a) has a myopic patient benefit goal also subject to a power constraint.

## 3 Established Views on RAR

In this section, we critically examine some published views on RAR. We present them labeled as questions because we have received them as such during informal exchanges with trial statisticians. We provide a complementary view of the use of RAR procedures, which acknowledges problems and disadvantages, but also emphasizes the solutions and advantages.

In what follows, we base our discussion on specific examples of RAR procedures only as a way to illustrate how some established views on RAR do not hold in general. The examples used below are by no means presented as the ‘best’ RAR procedures, or even necessarily recommended for use in practice − such judgments critically depend on the context and goals of the specific trial under consideration. We direct the reader to Section 4 for the latter point.

### 3.1 Does RAR lead to a substantial chance of allocating more patients to an inferior treatment?

Thall et al. (2016) give a number of undesirable properties of RAR, including the following:

…there may be a surprisingly high probability of a sample size imbalance in the wrong direction, with a much larger number of patients assigned to the inferior treatment arm, so that [RAR] has an effect that is the opposite of what was intended.

In simulation studies of two-arm trials with a binary outcome in Thall et al. (2015, 2016) TS is shown to have a substantial chance (up to 43% for the parameter values considered) of producing sample size imbalances in the wrong direction (i.e. the inferior arm) of more than 20 patients out of a maximum of 200. While this result holds for the specific BRAR procedure in the scenarios under consideration in that work, these conclusions do not hold for all types of RAR. These authors were among the first to compute this metric of sample size imbalance, and most of the RAR literature does not report it (or related ones). Hence it is unclear how other families of RAR procedures perform in this regard. To address this, we perform a new simulation study in the two-arm trial setting with a binary outcome. We compare the following range of RAR procedures:

- *Permuted block randomization [PBR]*: patients are randomized in blocks to the treatments so that exact balance is achieved for each block (and hence at the end of the trial).
- *Thall and Wathen [TW(c)]*: randomizes patient *i* to treatment *k* = 1 with probability
  $$\pi_{1,i}=\frac{{\left[P\left(p_{1}>p_{0}|a^{i-1},Y^{i-1}\right)\right]}^{c}}{{\left[P\left(p_{1}>p_{0}|a^{i-1},Y^{i-1}\right)\right]}^{c}+{\left[1-P\left(p_{1}>p_{0}|a^{i-1},Y^{i-1}\right)\right]}^{c}}$$
  Here *P*(*p*_1_ > *p*_0_ | ***a***^*i*−1^, ***Y***^*i*−1^) is the posterior probability that the experimental treatment has a higher success rate than the control treatment. The parameter *c* controls the variability of the procedure. Setting *c* = 0 gives ER, while setting *c* = 1 gives TS as described in Section 2.3. Thall and Wathen (2007) suggest setting *c* equal to 1/2 or *i*/(2*n*).
- *Randomized Play-the-Winner Rule [RPW]*: see Section 2.1 and Wei and Durham (1978).
- *Drop-The-Loser rule [DTL]*: a generalization of the RPW proposed by Ivanova (2003).
- *Doubly-adaptive Biased Coin Design [DBCD]*: a response-adaptive procedure targeting the optimal ratio of Rosenberger et al. (2001a). For details, see Hu and Zhang (2004a).
- *Efficient Response-Adaptive Randomization Designs [ERADE]*: a response-adaptive procedure targeting the optimal allocation ratio of Rosenberger et al. (2001a). It attains the lower bound of the allocation variances, see Hu et al. (2009) for further details.
- *Forward-looking Gittins Index [FLGI(b)]*: a RAR procedure with near-optimal patient benefit properties proposed in Villar et al. (2015b). This depends on a block size *b*.
- *Oracle*: hypothetical non-randomized rule that assigns all patients to the *true* best-performing arm (i.e. *π*_k*,*i*_ = 1 for k* = max_*k*_ *p_k_* and *π_k,i_* = 0 otherwise for all *i*).

In our simulations, we initially set *p*_0_ = 0.25 and vary the values of *p*_1_ (with *p*_1_ > *p*_0_) and *n*. Unlike in Thall et al. (2016), we do not include early stopping in order to isolate the effects of using RAR procedures. We evaluate performance in terms of several imbalance metrics including *E*(*N*_1_ − *N*_0_) and the (2.5 percentile, 97.5 percentile) of (*N*_1_ − *N*_0_); the probability of a imbalance of more than 10% of the total sample size in the wrong direction (i.e. allocating more patients to the inferior arm), denoted *Ŝ*_0.1_ = Pr(*N*_0_ > *N*_1_ +0.1*n*) when *p*_1_ > *p*_0_; the ENS and its standard deviation. Note that our measure of *Ŝ*_0.1_ coincides with the single imbalance measure used in Thall et al. (2016) when *n* = 200.

Table 1 shows the results for *p*_1_ = 0.35 and *n* ∈ {200, 654}. When *n* = 200, TS has a substantial probability (*Ŝ*_0.1_ ≈ 14%) of an undesirable imbalance in the wrong direction, while using the Thall and Wathen (TW) procedure reduces this probability, which (as expected) agrees with Thall et al. (2016). Unsurprisingly, the bandit-based procedures (i.e. FLGI) also has relatively large values of *Ŝ*_0.1_, although interestingly these are still smaller than for TS which could be due to their non-myopic nature. Meanwhile, ER has *Ŝ*_0.1_ ≈ 0.07, which provides a simple theoretical baseline (although in practice, for larger trials a form of PBR would be most suitable). In contrast, the RPW, DBCD, ERADE and DTL procedures all have values of *Ŝ*_0.1_ of 0.01 or less, which is also reflected in the ranges for the sample size imbalance. These procedures are hence comparable to PBR in terms of this imbalance metric.

The total sample size (in comparison to the treatment effect) can have a large impact on these imbalance metrics. When *n* = 200, the trial has low power to declare the experimental treatment superior to the control. If the sample size is chosen so that ER yields a power of at least 80% (when using the standard *Z*-test), then we require *n* ≥ 654. For *n* = 654, Table 1 shows that the values of *Ŝ*_0.1_ are substantially reduced for TS, the TW procedure and the bandit-based procedures. The ranges for *N*_1_ − *N*_0_ suggest that TS and the bandit-based procedures still have a small risk of getting ‘stuck’ on the wrong treatment.

**FIG 2:**
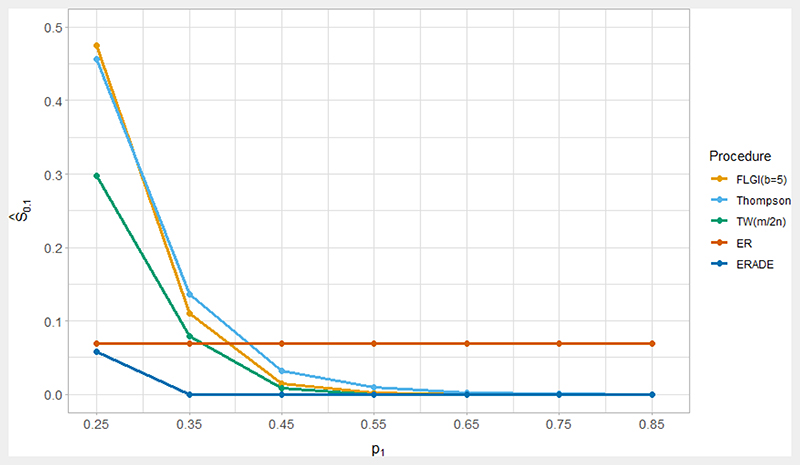
*Plot of Ŝ*_0.1_ *for various RAR procedures as a function of p*_1_, *where p*_0_ = 0.25 *and n* = 200. *Each data point is the mean of* 10^4^ *trial replicates*.

Another important factor is the magnitude of the difference between *p*_0_ and *p*_1_ or the treatment effect. The scenario considered above with *p*_0_ = 0.25 and *p*_1_ = 0.35 is a relatively small difference (as shown by the large sample size required to achieve a power of 80%), and the more patient-benefit oriented rules would not perform well in terms of sample size imbalance in this case. Table A2 (in the Appendix) shows the results when *p*_1_ = 0.45 and *n* = 200. The values of *Ŝ*_0.1_ are substantially reduced for TS as well as for the TW and bandit-based procedures, being much less than for ER and not substantially greater than using PBR. In terms of the mean and ranges for *N*_1_ − *N*_0_, these are now especially appealing for FLGI. Figure 2 extends this analysis by considering the value of *Ŝ*_0.1_ for a range of values of *p*_1_ from 0.25 to 0.85 when *n* = 200 to illustrate how this issue evolves as we move away from the null hypothesis scenario (while recognising that small differences of *p*_1_ from *p*_0_ may not be practically important). For *p*_1_ greater than about 0.4, the probability of a substantial imbalance in the wrong direction is higher for a simple ER design than for all of the other RAR procedures considered.

Figure 2 demonstrates another issue of *Ŝ*_0.1_ as a performance measure. This probability of imbalance increases for the RAR procedures considered as the difference *p*_1_ − *p*_0_ decreases, but as this difference decreases, so do the consequences of assigning patients to the inferior treatment. Table 1 depicts trade-offs between sample size imbalance (as measured by *N*_1_ − *N*_0_ and *Ŝ*_0.1_) and the ENS. The most patient-benefit oriented RAR procedures (TS and FLGI) have the highest ENS, which are in fact close to the highest possible ENS (the ‘Oracle’ procedure). However, these procedures also perform the worst in terms of sample size imbalance. This demonstrates our general point that careful consideration is needed by looking at a variety of performance measures instead of focusing on a single measure such as *Ŝ*_0.1_.

#### Summary

In summary, RAR procedures do not necessarily have a high probability of a substantial sample size imbalance in the wrong direction, when compared with using ER or PBR. This probability crucially depends on the true treatment effect, as well as the planned sample size of the trial. These results suggest that sample size imbalance may be larger when the effect size is smaller (i.e. being close to the null), and we hypothesize that this may generalize beyond the binary context.

If sample size imbalance is of particular concern in a specific trial context, an option is to consider the use of constraints to avoid imbalance, such as the constrained optimization approach of Williamson et al. (2017). Recently Lee and Lee (2021) also proposed an adaptive clip method (i.e. having a lower bound on the allocation probabilities) that can be used in conjunction with BRAR to reduce the chance of imbalance. Potential sample size imbalances need to be carefully evaluated in light of other performance metrics: restricting imbalance limits the potential for the patient benefit gains RAR can attain. Of course, if sample size imbalance needs to be strictly controlled in a trial, a restricted randomization scheme (such as PBR) may be more appropriate than using RAR.

### 3.2 Does the use of RAR reduce statistical power?

Perhaps one of the most well established views about RAR procedures is that their use reduces statistical power, as stated in Thall et al. (2015):

Compared with an ER design, [RAR] …[has] smaller power to detect treatment differences.

Similar statements appear in Korn and Freidlin (2011a) and Thall et al. (2016). Through simulation studies, these papers (all focused on the two-arm setting with binary outcomes) show that ER can have a higher power than BRAR for a fixed sample size, or equivalently that a larger sample size is needed for BRAR to achieve the same power and type I error rate as an ER design.

These papers only consider the BRAR procedure proposed by Thall and Wathen (2007) (see Section 3.1 for a formal definition). As shown in Hu and Rosenberger (2006), RAR procedures will have additional variability introduced by the correlation between the outcome *Y_k,i_* and allocation *a_k,i_*, and this will in turn translate into a higher variability var(*T_n_*) of a statistical test *T_n_* (hence reducing power). Yet, as we discuss, there exist RAR procedures that control for this, so that their use does not necessarily reduce power. In this section, we focus solely on power considerations and we assume the use of standard (frequentist) inferential tests to make power comparisons, which we return to in Section 3.3. Finally, we present the two-arm and multi-arm trial settings in distinct subsections below, since (as discussed in Section 1.2) the definition of ‘power’ becomes more complex in the latter setting.

#### Two-arm trials

Some RAR procedures formally target optimality criteria as a reflection of the trial’s objectives, including power. In a binary outcome setting, as in Rosenberger and Hu (2004) with the *Z*-test given in equation (3) and defining *ρ* = *N*_1_*/n*, one strategy is to fix the power of the trial and find (*N*_0_, *N*_1_) to minimize the total sample size *n*. This is equivalent to fixing *n* and finding (*N*_0_, *N*_1_) to maximize the power. This gives the optimal ratio known as Neyman allocation, $\rho_{\text{Neyman}}^{*}$:

(5) $$\rho_{\text{Neyman}}^{*}=\frac{\sqrt{p_{1}\left(1-p_{1}\right)}}{\sqrt{p_{0}\left(1-p_{0}\right)}+\sqrt{p_{1}\left(1-p_{1}\right)}}.$$

In general, $\rho_{\text{Neyman}}^{*}\neq 1/2$ and hence ER does not maximize the power for a given *n* when responses are binary. The notion that ER maximises power in general is an established belief that appears in many papers (see e.g. Torgerson and Campbell (2000)) but it only holds in specific settings (e.g. if comparing means of two normally-distributed outcomes with a common variance).

An ethical problem with this allocation maximising power is that if *p*_0_ + *p*_1_ > 1, more patients will be assigned to the treatment with the smaller *p_k_*. This shows the potential trade-off between power and patient benefit and motivated the alternative approach by Rosenberger et al. (2001a) as in Section 2.3 − see equation (4). The optimal solution $\rho_{R}^{*}$ is as follows:

(6) $$\rho_{R}^{*}=\frac{\sqrt{p_{1}}}{\sqrt{p_{0}}+\sqrt{p_{1}}}.$$

**FIG 3:**
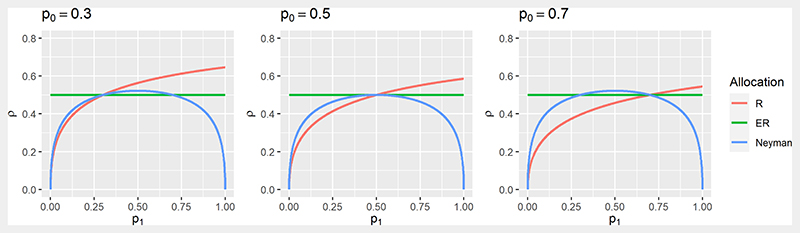
*Plot of the optimal allocation ratios* $\rho_{Neyman}^{*}$ *and* $\rho_{R}^{*}$ *as a function of p*_1_, *for p*_0_ ∈ {0.3, 0.5, 0.7}.

Figure 3 shows the optimal allocation ratios $\rho_{\text{Neyman}}^{*}$ and $\rho_{R}^{*}$ as a function of *p*_1_ for different values of *p*_0_. Both coincide with ER only when *p*_1_ = *p*_0_ while $\rho_{R}^{*}$ always allocates more patients to the treatment which has the higher success rate. Looking at $\rho_{\text{Neyman}}^{*}$, for *p*_1_ + *p*_0_ < 1 a higher allocation to the treatment with the higher success rate will be more powerful than ER.

For many types of endpoints, such as binomial and survival outcomes, the model parameters in the optimization problem are unknown and need to be estimated from the accrued data. These estimates can then be used (for example) with DBCD (Hu and Zhang, 2004a) or ERADE (Hu et al., 2009) to target the optimal allocation ratio. Using the DBCD in this manner, Rosenberger and Hu (2004) found in their simulation studies that it was

…as powerful or slightly more powerful than complete randomization in every case and expected treatment failures were always less

Similar theoretical results are in Yuan and Yin (2011). This is consistent with a general guidelines given by Hu and Rosenberger (2006) for using RAR procedures in a clinical trial, one of which is that *power should be preserved*. RAR procedures that achieve this aim have been derived (in a similar spirit to the optimal allocation above) for continuous (Zhang and Rosenberger, 2006) and survival (Zhang and Rosenberger, 2007) outcomes. Another line of work by Baldi-Antognini et al. (2018a,b) has looked at modifying the classical Wald test statistic for normally distributed outcomes in order to simultaneously improve power and patient benefit.

#### Multi-arm trials

Similar concerns about ‘power’ for multi-arm RAR procedures have been discussed. For example, Wathen and Thall (2017) simulate a variety of five-arm trial scenarios and conclude

In multi-arm trials, compared to ER, several commonly used adaptive randomization methods give much lower probability of selecting superior treatments.

Similarly, Korn and Freidlin (2011b) simulate a four-arm trial and find that a larger average sample size is needed when using a RAR procedure instead of ER in order to achieve the same marginal power. As discussed in Section 1.2, there are different power definitions in this case. Lee et al. (2012) reach similar conclusions in the three-arm setting for disjunctive power. However, all these papers only consider variants of the TW procedure (the “commonly used adaptive randomization methods” quoted above) for multi-arm trials, and these conclusions may not hold for RAR procedures in general.

The optimal allocation in Rosenberger et al. (2001a) can be generalized for multi-arm trials, assuming a global null hypothesis. The allocation is optimal in that it fixes the power to reject the global null and minimizes the ENF. This was first derived by Tymofyeyev et al. (2007), who showed through simulation that for three treatment arms, using the DBCD to target the optimal allocation

…provides increases in power along the lines of 2–4% [in absolute terms]. The increase in power contradicts the conclusions of other authors who have explored other randomization procedures [for two-arm trials]

Similar conclusions are given in Jeon and Hu (2010), Sverdlov and Rosenberger (2013a) and Bello and Sabo (2016).

These optimal allocation procedures maintain (or increase) the power of the test to reject the global null, but may have low marginal powers compared with ER in some scenarios, as shown in Villar et al. (2015b). However, even considering the marginal power to reject the null hypothesis 𝓗_0,*k*_⋆: *θ_k_*⋆ = *θ*_0_ for the best treatment k*, Villar et al. (2015b) propose non-myopic RAR procedures (i.e. the “controlled” FLGI rules) that in some scenarios have both a higher marginal power and a higher ENS when compared with ER with the same sample size.

Finally, the power comparisons made throughout this section have been against ER. A different comparison would be against group-sequential and Multi-Arm Multi-Stage (MAMS) designs using ER in each stage. Both Wason and Trippa (2014) and Lin and Bunn (2017) show that BRAR can have a higher power than MAMS designs when there is a single effective treatment. More recently, Viele et al. (2020a) show that the control allocation plays a part in achieving the power of a study when a variant of the TW procedure is implemented. These authors also explore other design aspects in conjunction with the control allocation, and find that RAR can have acceptable power in some settings (Viele et al., 2020b).

#### Summary

In conclusion, if RAR is used to improve patient benefit properties (in terms of ENF or ENS), then the power compared to ER can be preserved through an appropriate choice of the RAR procedure for the trial setting. Of course, this needs to be made with the objectives of the trial in mind (see Section 4). If maximizing power is a key objective, then using ER (instead of RAR) may not necessarily achieve this, even for two-arm trials. As discussed above, the nature of the response distribution plays an important role in these considerations, with much of the RAR literature focusing on binary responses.

### 3.3 Does RAR make valid statistical inference (more) challenging?

The Bayesian approach to statistical inference allows the seamless analysis of results of a trial that uses RAR. However, as noted in Proschan and Evans (2020),

The frequentist approach faces great difficulties in the setting of RAR …Use of RAR eliminates the great majority of standard analysis methods …

Rosenberger and Lachin (2016) comment on the reason for this:

Inference for [RAR] is very complicated because both the treatment assignments and responses are correlated.

This raises a key question: how can an investigator validly analyze a trial using RAR in a frequentist framework? In terms of the notation in Section 1.1, this can be formalized as determining whether standard test statistics *T_n_* can be relied on for hypothesis testing (i.e. without inflation of type I error rates), and whether standard estimators ${\hat{\theta }}_{k}$ are biased (and if so, by how much). Such questions are important for adaptive trial designs in general and not only for those using RAR. The challenge of statistical inference (within the frequentist framework) is naturally still seen as a key barrier to the use of RAR in clinical practice. We next discuss how valid statistical inference, especially in terms of type I error rate control and unbiased estimation, is possible for a wide variety of RAR procedures. Note that in what follows, we do not consider time trends and patient drift, as a separate discussion is given in Section 3.4.

#### Asymptotic inference

A straightforward approach to frequentist inference for a trial using RAR is to use standard statistical tests and estimators *without* adjustment. This is justified by asymptotic properties that hold for a large class of RAR procedures, including in the multi-arm setting. Firstly, Melfi and Page (2000) proved that an estimator ${\hat{\theta }}_{k}$ that is consistent (i.e. ${\hat{\theta }}_{k}\to \theta_{k}$ as the sample size *n* → ∞) when *Y_k,i_* are independent and identically distributed will also be consistent for any RAR procedure for which *N_k_* → ∞.

Secondly, Hu and Rosenberger (2006) showed that when responses *Y_k,i_* follow an exponential family, simple conditions on the RAR procedure ensure the asymptotic normality of the MLE. The condition is that the allocation proportions for each arm $\sum_{i=1}^{n}1\left\{a_{i,k}=1\right\}/n\to \rho$, where *ρ* ∈ (0, 1). This implies that the RAR procedure cannot ‘select’ a treatment during the trial by having allocation probabilities tending to 1 or 0. Since many test statistics are functions of the MLE, this also implies that the asymptotic null distribution of such test statistics is not affected by the RAR. Furthermore, if a given RAR procedure does not have this property, then there is a straightforward modification to ensure it holds by bounding (or ‘clipping’) the allocation probabilities *π_i,k_*, see Baldi-Antognini et al. (2022a). These asymptotic results are the justification for the first guideline given by Hu and Rosenberger (2006) on RAR procedures, which states that “Standard inferential tests can be used at the conclusion of the trial.”

#### Finite sample inference

The validity of asymptotic results to use standard tests and estimators requires a sufficiently large sample size, and the effect of a smaller sample size on inference is greater the more imbalanced the RAR procedure is (e.g. see the results in Williamson and Villar (2020)). As noted by Rosenberger et al. (2012), for some RAR procedures in a two-arm setting, there is extensive literature on the accuracy of asymptotic approximations under moderate sample sizes using simulations (Hu and Rosenberger, 2003; Rosenberger and Hu, 2004; Zhang and Rosenberger, 2006). For the DBCD, sample sizes of *n* = 50 to 100 are sufficient, while for urn models reasonable convergence is achieved for *n* = 100. For these procedures, Gu and Lee (2010) explored which asymptotic test statistic to use for a clinical trial with a small to medium sample size and binary responses.

When the asymptotic results above cannot be used, either because of small sample sizes or because the conditions on the RAR procedures are not met, then alternative methods for testing and estimation have been proposed. We summarize the main methods below, concentrating on type I error rate control and unbiased estimation.

A common method for controlling the type I error rate, particularly for BRAR procedures, is a simulation-based calibration approach, see e.g. see the FDA guidance on simulations for adaptive design planning (FDA, 2019, Section VI.A). Given a trial design that uses RAR and an analysis strategy, a large number of trials are simulated under the null. Applying the analysis strategy to each of these trial realizations gives a Monte Carlo approximation of the relevant error rates (see Section 1.2). If necessary, the analysis strategy can be adjusted to satisfy type I error constraints. Variations of this approach have been used in Wason and Trippa (2014); Wathen and Thall (2017); Baldi-Antognini et al. (2022b). These approaches can be computationally intensive, and there are no guarantees beyond the parametric space explored in the simulations.

A related approach is to use a *re-randomization* test, also known as *randomization-based inference*. In such a test, the outcomes ***y***^(*n*)^ are taken as fixed, but the allocations ***a***^(*n*)^ are regenerated many times using the RAR procedure under the null hypothesis. For each replicate, the test statistic *T_n_* is recalculated, and a consistent estimator of the *p*-value is given by the proportion of test statistics that are at least as extreme as the value actually observed. Intuitively, this is valid because under the null hypothesis of no treatment differences, ***y***^(*n*)^ and ***a***^(*n*)^ are independent. Simon and Simon (2011) give general conditions under which the re-randomization test guarantees the type I error rate for all RAR procedures. Galbete and Rosenberger (2016) showed that 15, 000 replicates are sufficient to accurately estimate even very small *p*-values. An advantage of re-randomization tests is that they protect against *unknown* time trends (see Section 3.4). However, re-randomization tests can suffer from a lower power compared with using standard tests (Villar et al., 2018), particularly if the RAR procedure has allocation probabilities that are highly variable (Proschan and Dodd, 2019).

The implementation of these methods may lead to computational cost and Monte Carlo error concerns. There have been a few proposals that do not rely on simulations. Robertson and Wason (2019); Glimm and Robertson (2022) proposed a re-weighting of the usual *Z*-test that guarantees familywise error control for a large class of RAR procedures for multi-arm trials with normally-distributed outcomes, although with a potential loss of power. Galbete et al. (2016) derived the exact distribution of a test statistic for a family of RAR procedures in the context of a two-arm trial with binary outcomes, and hence showed how to obtain exact *p*-values.

Turning now to estimation bias, the MLEs for the parameters of interest for a trial using RAR will typically be biased in small samples. This is illustrated for a number of RAR procedures for binary outcomes through simulation in Villar et al. (2015a); Thall et al. (2015). However, the latter point out that in their setting, which incorporates early stopping,

…most of the bias appears to be due to continuous treatment comparison, rather than AR *per se*.

Hence it is important to distinguish bias induced by early stopping from that induced by the RAR procedure. In a binary setting and for multi-arm RAR procedures without early stopping, the bias of the MLE ${\hat{p}}_{k}$ is given in Bowden and Trippa (2017):

(7) $$\text{bias}\left({\hat{p}}_{k}\right)=E\left({\hat{p}}_{k}\right)-p_{k}=-\frac{\text{Cov}\left(N_{k},{\hat{p}}_{k}\right)}{E\left(N_{k}\right)}.$$

In a typical RAR procedure that assigns more patients to treatments that appear superior (i.e. $\text{Cov}\left(N_{k},{\hat{p}}_{k}\right)>0$), equation (7) shows the bias of the MLE is negative. The magnitude of this bias is decreasing with the expected number of patients assigned to the treatment (i.e. as *E*(*N_k_*) → ∞). When estimating the treatment *difference* however, the bias can be either negative or positive, which agrees with the results in Thall et al. (2015).

Bowden and Trippa (2017) showed that if there is no early stopping, the magnitude of the bias tends to be small for the RPW rule and the BRAR procedure proposed by Trippa et al. (2012). For more imbalanced RAR procedures, the bias can be larger however, e.g. see Williamson and Villar (2020). As a solution, Bowden and Trippa (2017) proposed using inverse probability weighting and Rao-Blackwellization to produce unbiased MLEs, although these can be computationally intensive. For urn-based RAR procedures, Coad and Ivanova (2001) also proposed bias-corrected estimators. For sequential maximum likelihood procedures and the DBCD, Wang et al. (2020) evaluate the bias issue and propose a solution. Meanwhile, Marschner (2021) proposed a general framework for analysing adaptive experiments, included trials using RAR, and explored the merits of both conditional and unconditional estimation.

Finally, adjusted confidence intervals for RAR procedures have received less attention in the literature. Rosenberger and Hu (1999) proposed a bootstrap procedure for multi-arm RAR procedures with binary responses, while Coad and Govindarajulu (2000) proposed corrected confidence intervals for a sequential adaptive design in a two-arm trial with binary responses. Recently, Hadad et al. (2021) proposed a strategy to construct asymptotically valid confidence intervals for a large class of adaptive experiments (including RAR).

#### Summary

For trials with sufficiently large sample sizes, asymptotic results justify the use of standard tests and frequentist inference procedures when using many types of RAR. When asymptotic results do not hold, inference does become more challenging compared with using ER but it is possible. There is a growing body of literature demonstrating how a trial using RAR, if designed and analyzed appropriately, can control the type I error rate and correct for the bias of the MLE. All this should give increased confidence that the results from a trial using RAR can be both valid and convincing. We reiterate that from a Bayesian viewpoint, the use of RAR does not pose additional inferential challenges.

### 3.4 Does using RAR make robust inference difficult if there is potential for time trends?

The occurrence of time trends caused by changes in the standard of care or by patient drift (i.e. changes in the characteristics of recruited patients over time) is seen as a major barrier to the use of RAR in practice

One of the most prominent arguments against the use of [RAR] is that it can lead to biased estimates in the presence of parameter drift. (Thall et al., 2015)

A more fundamental concern with adaptive randomization, which was noted when it was first proposed, is the potential for bias if there are any time trends in the prognostic mix of the patients accruing to the trial. In fact, time trends associated with the outcome due to any cause can lead to problems with straightforward implementations of adaptive randomization. (Korn and Freidlin, 2011a)

Both papers cited above show (for BRAR procedures) that time trends can substantially inflate the type I error rate when using standard analysis methods, and induce bias into the MLE. Further simulation results are given in Jiang et al. (2020). Villar et al. (2018) present a simulation study for different time trend assumptions and a variety of RAR procedures in trials with binary outcomes including the multi-arm setting.

As an illustrative numeric example from Villar et al. (2018), consider a two-arm trial with binary outcomes, where *n* = 100 and patients are randomized in groups of size 10. Suppose there is a linear upward trend in *p*_0_, so that the overall time trend within the trial

$$D=\mathrm{Pr}\left(Y_{0,i}=1|90<i\leq 100\right)-Pr\left(Y_{0,i}=1|0<i\leq 10\right)$$

varies in *D* ∈ {0, 0.01, 0.02, 0.04, 0.08, 0.16, 0.24}. In this case, under the null scenario where *p*_0_ = *p*_1_ at all time points, the optimal allocation of Rosenberger et al. (2001a) has an almost constant type I error rate, just above the nominal 0.05 level. The TW procedure (Thall and Wathen, 2007) has an inflated type I error rate (about 0.09) even without any time trend (i.e. *D* = 0), which increased to almost 0.15 when *D* = 0.24. Finally, the patient-benefit oriented FLGI rule (Villar et al., 2015b) has a type I error rate going from 0.05 to almost 0.25 as *D* increased from 0 to 0.24. These results show that for RAR procedures, even changes in just *p*_0_ (or *p*_1_) over time can have a considerable impact on operating characteristics. Hence time trends in the treatment effect (however defined) will also be expected to have similar impacts.

Although time trends can inflate the type I error when using RAR procedures, there are two important caveats given in Villar et al. (2018). Firstly, certain power-oriented RAR procedures appear to be effectively immune to the time trends considered in their paper. In particular, RAR procedures that protect the allocation to the control arm are particularly robust. A possible explanation is that those rules have a smaller imbalance, as suggested in Baldi-Antognini et al. (2022a). Secondly, as discussed in Villar et al. (2018), a largely ignored but highly relevant issue is the size of the trend and its likelihood of occurrence in a specific trial:

…the magnitude of the temporal trend necessary to seriously inflate the type I error of the patient benefit-oriented RAR rules need to be of an important magnitude (i.e. change larger than 25% in its outcome probability) to be a source of concern.

A more general issue around time trends is that they can invalidate the key assumption that observations about treatments are exchangeable (i.e. that subjects receiving the same treatment arm have the same probability of success). This, in turn, invalidates commonly used frequentist and Bayesian models, and hence the inference of the trial data. Type I error inflation and estimation bias can be seen as examples of this wider issue.

As Proschan and Evans (2020) put it, temporal trends are likely to occur in two settings:

…1) trials of long duration, such as platform trials in which treatments may continually be added over many years and 2) trials in infectious diseases such as MERS, Ebola virus, and coronavirus.

Despite this, little work has looked at estimating these trends, especially to inform trial design in the midst of an epidemic. Investigating these points is essential to make a sound assessment of the value of using RAR. A recent exception is in Johnson et al. (2022), where a two-arm vaccine trial for COVID-19 using RAR is studied using a model to simulate the epidemic (including linear trends).

As mentioned in Section 3.2, a robust method to prevent type I error inflation is to use a re-randomization test. Simulation studies illustrating the use of this test can be found in Galbete et al. (2016); Villar et al. (2018); Johnson et al. (2022). However, this can come at the cost of a considerably reduced power compared with using an unadjusted testing strategy. More recently, Wang and Rosenberger (2021) showed how to construct confidence intervals for randomization tests that are robust (in terms of coverage) to time trends.

An alternative to randomization-based inference is to use a stratified analysis. This was first proposed by Jennison and Turnbull (2000) for group-sequential designs, with subsequent work by Karrison et al. (2003); Korn and Freidlin (2011a). These papers show that a stratified analysis can eliminate the type I error inflation induced through time trends. However, Korn and Freidlin (2011a) also showed that this strategy can reduce the trial efficiency (see also Korn and Freidlin (2022) for similar arguments), both in terms of increasing the required sample size and the chance of patients being assigned to the inferior treatment.

Another approach is to explicitly incorporate time-trend information into the regression analysis. Jennison and Turnbull (2001) developed theory that allows the incorporation of polynomial time trends as covariates in a general normal linear regression model for group sequential designs, while Coad (1991) modified a class of sequential tests to incorporate a linear time trend for normally-distributed outcomes. Meanwhile, Villar et al. (2018) assessed incorporating the time trend into a logistic regression (for binary responses), and showed that this can alleviate type I error inflation if the trend is correctly specified However, this leads to a loss of power and complicates estimation (due to the technical problem of separation).

Finally, it is possible to try to control the impact of a time-trend *during* randomization. Rosenberger et al. (2001b) proposed a CARA procedure for a two-armed trial that can take a specific time trend as a covariate. More recently, Jiang et al. (2020) proposed a BRAR procedure that includes a time trend in a logistic regression model, and uses the resulting posterior probabilities as the basis for the randomization probabilities. This model-based procedure controls the type I error rate and mitigates estimation bias, but at the cost of reduced power.

#### Summary

Large time trends can inflate the type I error when using RAR, and this inflation becomes worse the more imbalanced the RAR procedure is. However, RAR procedures that protect the allocation to the control arm or impose restrictions to avoid extreme allocation probabilities are particularly robust. For other RAR procedures, analysis methods exist to mitigate the type I error inflation caused by time trends, although with a loss in power. Finally, we note that time trends can affect inference in all types of adaptive clinical trials, and not just those using RAR.

### 3.5 Is RAR more challenging to implement in practice?

In addition to the statistical aspects discussed in Sections 3.1−3.4, there are practical questions to consider to best implement RAR in the context of the study at hand. Most of these practical issues apply to other randomized designs (both adaptive and non-adaptive), so we focus here on a few that merit a specific discussion for RAR.

#### Measurement/classification error and missing data

Measurement error (for continuous variables) or classification error (for binary variables) and missing data are common in medical research. There are many approaches proposed to reduce the impact of these on statistical inference (see e.g. Guolo (2008); Little and Rubin (2002); Blackwell et al. (2017)) but very little literature on this in the context of RAR. The distinctive concern is that the sequentially updated allocation probabilities may be biased, and hence the design will not have its expected properties e.g. in terms of patient benefit.

A few articles looking at classification (or measurement) error in RAR include Li and Wang (2012), who derive optimal allocation targets under constant misclassification probabilities that differ between the arms, and Li and Wang (2013), who explore through simulation the effect of misclassification (in the two-arm setting) on optimal allocation designs.

As for missing data, Chen et al. (2022) consider the performance of BRAR procedures under the assumption of missing at random (see Rubin (1976)) and with a single imputation for the missing responses. They found that these procedures encourage more assignments in the arm with missing data, and that simple mean imputation can largely mitigate this effect. Williamson and Villar (2020) propose an imputation method for a bandit-based RAR when the outcome is undefined. Incomplete data for such extreme cases is imputed with random samples drawn from the tails of the distribution. Simulations suggest that imputing in this way is better than ignoring missingness in terms of patient benefit and other metrics. More complex scenarios, e.g. data not missing at random, remain unexplored, but this is the case for adaptive trials in general except for some simple settings (see e.g. Lee et al. (2018)).

#### Delayed responses and recruitment rate

The use of RAR is not feasible if the patient outcomes are only observed after all patients have been recruited and randomized. This is rare but may happen if the recruitment period is short (e.g. due to a high recruitment rate), or when the outcome of interest takes a long time to observe. One way to address the latter is to use a surrogate outcome that is more quickly observed as for example in Tamura et al. (1994). Another possibility is to use a randomization plan that is implemented in stages as more data becomes available (like for FLGI).

In general, as stated in Hu and Rosenberger (2006, pg. 105):

From a practical perspective, there is no logistical difficulty in incorporating delayed responses into the RAR procedures, provided some responses become available during the recruitment and randomization period.

However, statistical inferences at the end of the trial can be affected. This is explored theoretically for urn models (Bai et al., 2002; Hu and Zhang, 2004b; Zhang et al., 2007) as well as the DBCD (Hu et al., 2008). These papers show that the asymptotic properties of these RAR procedures are preserved under widely applicable conditions. In particular, when more than 60% of responses are available by the end of the recruitment period, simulations show that the power of the trial is essentially unaffected.

#### Patient consent to be randomized

Patient consent protects patients’ autonomy, and requires an appropriate balance between information disclosure and understanding (Beauchamp, 1997). There is evidence that the basic elements to ensure informed consent (recall and understanding) can be difficult to ensure even for non-adaptive studies (Sugarman et al., 1999; Dawson, 2009). The added complexity of allocation probabilities that may change in response to accumulated data only makes achieving patient consent more challenging. Moreover, since these novel adaptive procedures are still rarely used, there is little practical experience to draw upon.

#### Implementing randomization changes during a study

Randomization of patients, whether adaptive or not, must be done in accordance with standards of good clinical practice. As such, in most clinical trials randomization is done through a dedicated and secure web-based system that is available 24/7. In the UK, for example, most clinical trials units will outsource their randomization to external companies. This outsourcing is practical but costly, and limits the ways in which randomization can be implemented to those currently offered by such companies. To the best of the authors’ knowledge, in the UK common providers treat every change in a randomization ratio as a trial change (which is charged as such), rather than being considered an integral part of the trial design. Beyond the extra costs and limitations to the use of RAR that this brings, it also introduces unnecessary delays as randomization is stopped while the change is implemented.

A related issue is that of preserving treatment blinding, which is key to the integrity of clinical trials. This is particularly important when using RAR, as if an investigator knows which treatment is more likely to be allocated next, selection bias is more likely to occur. In most cases, preserving blindness will require an independent statistician (which requires extra resources) to handle the interim data and implement the randomization, or a data manager can provide data to an external randomization provider who can then update the randomization probabilities independently of the clinical and statistical team. Further discussion on these issues can be found in Sverdlov and Rosenberger (2013b).

### 3.6 Is using RAR in clinical trials (more) ethical?

Ethical reasons are the most cited arguments in favor of using RAR to design clinical trials.

Our explicit goal is to treat patients more effectively, but a happy side effect is that we learn efficiently. (Berry, 2004)

Research in [RAR] developed as a response to a classical ethical dilemma in clinical trials. (Hu and Rosenberger, 2006)

Nevertheless, there are also arguments that RAR may not be ethically preferred.

For RCTs [Randomised Controlled Trials] where treatment comparison is the primary scientific goal, it appears that in most cases designs with fixed randomization probabilities and group sequential decision rules are preferable to AR [RAR] scientifically, ethically and logistically (Thall et al., 2016)

Clinical research poses several ethical challenges. There is an inevitable tension between clinical research and clinical practice, as the latter is concerned with best treating an individual patient while the former is focused on ‘future’ patients. Clinical research is associated with a clinical trial whose main aims are the testing and estimation goals as in Section 1.2. Clinical practice is directly concerned with patient benefit goals which are, at best, secondary aims in traditional clinical trials. Such ethical questions are becoming more discussed as personalized treatment becomes more embedded into research, as is the case for oncology (London, 2018).

Although treating patients in the trial “more effectively” using RAR appears to be ethically attractive, particularly from the recruited patients’ perspective, the extent to which these and other adaptive designs are more “ethical” than traditional designs is only starting to be addressed by ethicists. Thus, we do not aim to answer the question whether RAR is (more) ethical or not, as this requires a specific answer for each method and trial context. Instead, we review key concepts that could affect this answer and that come from formal discussions by ethicists.

#### The “equipoise” concept and the ethical grounds for randomizing patients

Equipoise is typically defined as a state of uncertainty of the individual investigator regarding the relative merits of interventions for a population of patients. Such uncertainty justifies randomizing patients to treatments as this does not imply knowingly disadvantaging patients. This concept may extend to include “honest, professional disagreement among expert clinicians” about the relative merits of interventions (Freedman, 1987). This broader definition is known as ‘clinical equipoise’ while the former is ‘theoretical equipoise’.

An argument against the use of RAR is that it violates the principle of equipoise on which clinical trials is based upon (Laage et al., 2017). Changing the randomization probabilities in light of patients’ responses may be viewed as breaking equipoise, because the updated allocation weights reflect the relative performance of the interventions in question. Once the randomization weights become unbalanced, the study has a preferred treatment and allocating participants to treatments regarded as inferior could be considered unethical. However, this argument that RAR is *unethical* because it breaks equipoise is based on two assumptions: 1) randomization ratios reflect a single agent’s beliefs about the relative merits of the interventions being tested; and 2) equipoise is a state of belief in which the relevant probabilities are assumed to be equally balanced. Neither of these two assumptions are consistent with the definition of ‘clinical equipoise’ as the clinical community is multi-agent and disagreement among these agents will not necessarily correspond to a 50%-50% split of opinions.

#### Patient horizon (individual and collective ethics)

The ethical value of RAR (and of other trial designs) depends directly on the trial’s specific aim in relation to its context. For example, a feature that considerably affects comparisons of design options is disease prevalence (a concept linked to that of patient horizon (Anscombe, 1963; Colton, 1963)). Suppose a clinical trial is being planned where *T* denotes the “patient horizon” for that study, i.e. those patients within and outside of the trial who will benefit from its conclusions. The exact value of *T* is never known but its order of magnitude considerably impacts the relative merits of competing trial goals. A trial relevant to patients with coronary artery disease will have the vast majority of the patient horizon outside of the trial, making the inferential goals of the study of paramount importance. On the other hand, a rare pediatric cancer is likely to have a large proportion of the patient population in the trial, heightening the tension between patient benefit and inferential goals. Similar considerations apply for emerging life-threatening diseases (e.g. the Ebola outbreak or the COVID-19 pandemic), where the patient horizon can be short for reasons other than prevalence. When the choice of design is based only on inferential considerations, there will be many instances in which a design may be considered inferior from a patient benefit viewpoint.

The impact of *T* on the ethical comparison of designs depends on considerations around individual and collective ethics and potential conflicts between these two. As Tamura et al. (1994, pg. 775) express it, RAR “represents a middle ground between the community benefit and the individual patient benefit” and because of this “it is subject to attack from either side”. This point has been well discussed and formally studied in the statistical literature (see Berry and Eick (1995); Cheng et al. (2003); Berry (2004)). Despite this, prevalence of a disease is almost never taken into account, neither in practice when designing trials nor in many methodological articles comparing RAR from an ethical point of view. See Lee and Lee (2021); Metelkina and Pronzato (2017) for recent attempts to address this.

#### Summary

We believe that the ethics of RAR needs more attention from ethicists, including collaborations between ethicists and statisticians to address the caveats and complexities of this broad family of methods. Positions based purely on statistical or ethical arguments in isolation are likely to be inadequate and arguments that involve ethical metrics should ideally be jointly discussed with multiple stakeholders. It is important to bear in mind that compromises between statistical and ethical objectives have very different implications under different settings. For example, the trade-offs between these two objectives may look very different in a two-arm trial setting compared to a multi-arm trial.

Ideally, how this interaction between ethics and statistics can proceed is as follows (as suggested by an anonymous reviewer). Ethics informs the relative importance of a trial’s goals, in particular the balance between individual and collective benefit. Once these priorities are in place, a statistical design that achieves these goals can be proposed. The ethical aspects can be revisited in light of the resulting properties of the statistical design. For example, suppose RAR is chosen to deliver a certain level of benefit to patients in the trial. This may require an increase in the trial size to preserve the inferential properties for future patients to be “ethical”. In that case, depending on the prevalence of the disease and the general context, a larger trial using the original RAR procedure may still deliver the most benefit to all patients and remain the preferred option. If this is not the case, then the ethics-design choice can be revisited.

## 4 Final Considerations and Discussion

The pace of methodological work on RAR and the debate over its use has certainly sped up in recent years, driven by the response to challenges during health crises like the COVID-19 pandemic and the increase uptake of these methods in machine learning and data science more generally. However, to some extent, the debate and methodological progress remain disconnected from each other. It is important to bear in mind that generalizations within such a large class of methods run the risk of being partial and misleading. Even for a single RAR procedure, its performance may vary considerably across the parameter space of interest. In this paper we have aimed to illustrate the breadth of RAR procedures by presenting a critical (but balanced) appraisal of well established views about RAR, and to help guide future research efforts towards areas that have received less attention.

We emphasize that this paper does not advocate for the use of RAR in all trial settings (but we also do not intend to discourage trialists from considering its use in general). There are contexts where other trial adaptations or even a fixed randomization design may be preferable for both methodological or practical reasons. This is important to consider with adaptive trials in general − sometimes it may be better to ‘keep it simple’ and use traditional non-adaptive designs instead (Wason et al., 2019). However, when the use of RAR is considered, it is helpful to remember that RAR encompasses a large set of possible design (and analysis) options, rather than being a homogeneous technique to either include or not. Indeed, many of the recent general criticisms and praise for RAR in clinical trials has been driven by arguments that apply to the particular subclass of BRAR, but may well not be as relevant for other RAR procedures.

Trade-offs in terms of different metrics are ubiquitous and in many cases unavoidable in clinical trials, as RAR procedures can address a specific need at the expense of a cost in a different area. Hence, a RAR procedure should be chosen carefully according to the specific context and goals of a trial, in light of the practical challenges and constraints that implementing RAR poses. Indeed, as noted by an anonymous reviewer, the approach of starting with a set of different RAR procedures and then choosing one based on comparing their performance as measured by different metrics is arguably going in the wrong direction. Instead, a preferable approach is to explicitly start by defining the type of trial and the investigators’ priorities in setting goals for the trial, and to then select a RAR design suited to these goals (see also Pitt (2021)).

Starting with the type of trial, factors such as the phase of clinical development, the number of treatment arms and the clinical endpoint will naturally influence the aims of the trial and the appropriateness of a design including RAR. Some types of clinical trial may be particularly suited to the use of a well-chosen RAR procedure − for example, in multi-arm trials it is natural to consider dropping poorly performing treatment arms, and RAR offers an intermediate option of reducing numbers on such treatments.

Given a particular type of trial, the aims of the trial can then be considered. Broadly speaking, these aims fall into two categories:

1) Determine how best to treat future patients after the trial concludes while avoiding (or minimizing) harm to patients in the trial;
2) Optimize treatment of patients in the trial itself (i.e. treat patients in the trial as effectively as possible).

Depending on the relative importance of these two, different RAR rules may be appropriate.

Once the aims of a study, their relative importance, and the corresponding metrics have all been agreed upon, the question of what an optimal RAR procedure is in terms of those metrics can be addressed. Ideally (as suggested by an anonymous editor), an optimal trial design can be found within this framework, rather than proposing ad hoc procedures and testing them against different metrics. However, in the literature reviewed we found the use of the term ‘optimal’ in relation to RAR procedures can have many different meanings. A broader definition of *optimality* may be beneficial to consider, not only including optimal allocation targets but also RAR families that have some other form of optimality (or near optimality). In any case, it is important to explicit say in what sense a procedure is ‘optimal’ when using this terminology.

As a general point (and one we more fully appreciate following recent discussions with applied trial statisticians), it is crucial to not consider statistical or methodological issues in isolation of practical issues. This may be key for the design of any experiment, but is more important for RAR and adaptive designs in general. For example, selection bias may be a big issue in some contexts, and if blinding is not possible, then the use of RAR may be less appropriate. Hence, greater collaboration and discussion between methodologists and applied trialists is useful to ensure that methods are developed with practical considerations in mind.

We would like to end with a short summary as to what we feel the future for RAR methods research should bring to improve its usefulness in clinical practice. We wrote this paper in a attempt to reconcile conflicting perspectives as much as to motivate researchers to address the issues mentioned here with new ideas. New work is needed to realise most of the potential advantages of RAR with fewer of its downsides while taking the trial context into account. With the increasing use of response-adaptive procedures in machine learning and data science more generally, this presents a golden opportunity for biostatisticians to embrace and lead the development of this wide adaptive class in both theory and practice.

As a general point, our hope is that any contribution to RAR methodology should be well contextualised within the ongoing debate in order to achieve practical impact and to avoid repeating common arguments that are already well-represented in the literature. When developing new proposals, it can be helpful to define terminology carefully, report a wide range of metrics and to be explicit about the potential limits of the conclusions made.

Firstly, we encourage the explicit definition and clear reporting of the metrics used to evaluate RAR procedures, as well as a broad look at multiple metrics (not just standard operating characteristics). For example, estimation and sample size imbalance metrics are relatively under-reported in the literature. Similarly, since many RAR procedures impact patient benefit, including at least one such metric (see Section 1.2) is useful when comparing RAR procedures.

Exploring a wide parametric space in simulations (and not only subsets of interest) can also be key. For example, Neyman allocation maximizes power, but for *p*_0_ + *p*_1_ > 1 assigns more patients to the inferior arm (see Section 3.2). Similarly, for the RPW rule the limiting distribution of the allocation proportion depends on whether *p*_0_ + *p*_1_ > 3/2 (Rosenberger and Lachin, 2016). Given the above, it is also important to discuss when certain properties may not apply to other RAR families. This could reduce the chances of readers misunderstanding the scope of conclusions about a specific family of RAR procedures. More generally, definitive statements based only on simulation results should be regarded with an appropriate degree of caution. There are no universal set of rules on how to conduct simulation studies (although useful guidelines are proposed by Morris et al. (2019)).

In terms of specific methodological research areas, a key open area is that of efficient and valid inference methods for RAR. As discussed in Section 3.3, a simple asymptotic approach for inference is valid in many case but it does not apply to all RAR procedures. On the other hand, valid methods for small samples (or time trends) such as randomization-based inference suffer from low power. Hence, the development of new inferential procedures for finite samples that do not suffer from a large loss in power would be very useful (as a recent example along these lines, see Barnett et al. (2021); Deliu et al. (2021)). For time trends in particular, there has been little work estimating the likelihood and magnitude of such trends in practice, especially in contexts such as emerging epidemics. More research would help to determine whether RAR would be appropriate for specific trial contexts.

Another open research question is how to account for missing data or measurement error when using RAR. Adjusted confidence intervals have also received little attention in the literature. More generally, further work is needed to expand the comparison of multi-arm RAR procedures (particularly in terms of different power definitions) beyond BRAR. In terms of design aspects, RAR has the under-explored potential for addressing delicate issues when designing studies with composite or complex endpoints. Another consideration is that block-randomized versions of RAR methods are much more likely to be applied in practice than fully sequential schemes, but open questions remain about how these implementations compare in terms of power and patient benefit. As well, it is still unclear in general how trial designs incorporating RAR compare with well-chosen group sequential and MAMS designs.

Finally, regardless of methodological considerations and future development, the use of RAR in practice would stil require the availability of user-friendly software for both the implementation of the randomization algorithm as well as for the analysis approaches that were mentioned in Section 3 of this paper.

## Supplementary Material

Supplementary Appendix

## Figures and Tables

**TABLE 1:** *Properties of various patient allocation procedures, where p*_0_ = 0.25 *and p*_1_ = 0.35. *Results are from* 10^4^ *trial replicates*.

*n*	Procedure	*N*_1_ − *N*_0_	*Ŝ*_0.1_	ENS
200	ER	0 (-28, 28)	0.069	60 (6.4)
(Low power)	PBR	0	0	60 (6.4)
	Oracle	200	0	70 (6.7)
	TS	95 (-182, 190)	0.137	65 (8.5)
	FLGI(*b* = 5)	114 (-176, 190)	0.111	66 (8.3)
	FLGI(*b* = 10)	115 (-172, 190)	0.100	66 (8.2)
	TW(1/2)	74 (-90, 174)	0.085	64 (7.5)
	TW(*i*/2*n*)	50 (-28, 122)	0.038	63 (6.8)
	RPW	14 (-16, 44)	0.011	61 (6.5)
	DBCD	17 (-10, 46)	0.003	61 (6.4)
	ERADE	16 (-6, 42)	0.000	61 (6.4)
	DTL	14 (-4, 32)	0.000	61 (6.6)
654	ER	0 (-50, 50)	0.005	196 (11.7)
(High power)	PBR	0	0	196 (11.6)
	Oracle	654	0	229 (12.2)
	TS	461 (-356, 640)	0.042	220 (17.0)
	FLGI(*b* = 5)	511 (-619, 645)	0.054	222 (18.5)
	FLGI(*b* = 10)	511 (-617, 645)	0.051	222 (18.0)
	TW(1/2)	384 (44, 594)	0.011	215 (14.2)
	TW(*i*/2*n*)	272 (54, 456)	0.010	210 (13.0)
	RPW	46 (-8, 100)	0.000	199 (11.8)
	DBCD	55 (8, 106)	0.000	199 (11.8)
	ERADE	54 (16, 96)	0.000	199 (11.7)
	DTL	46 (14, 80)	0.000	198 (11.7)

## Data Availability

Code to implement RAR algorithms given in Section 3.1 can be found at the end of the ‘Papers and code’ section at https://www.mrc-bsu.cam.ac.uk/software/miscellaneous-software/
