# Supplementary material for: Response-adaptive randomization in clinical trials: from myths to practical considerations
Source: Stat Sci. Author manuscript; Available in PMC 2023 Jun 14. (PMC7614644; doi:10.1214/22-STS865)
Supplement: Supplementary Appendix [file EMS153897-supplement-Supplementary_Appendix.pdf]

APPENDIX

TABLE A1
Table of acronyms used in the paper.

**Acronym**	**Definition**
BRAR	Bayesian Response-Adaptive Randomization
CARA	Covariate-Adjusted Response-Adaptive
DBCD	Doubly-adaptive Biased Coin Design
DTL	Drop-The-Loser
ENF	Expected Number of Failures
ENS	Expected Number of Successes
ER	Equal Randomization
ERADE	Efficient Response-Adaptive Randomization Designs
FLGI	Forward-Looking Gittins Index
MAMS	Multi-Arm Multi-Stage
MLE	Maximum Likelihood Estimator
PBR	Permuted Block Randomization
RAR	Response-Adaptive Randomization
RCT	Randomized Controlled Trial
RPW	Randomized Play-the-Winner
TS	Thompson Sampling
TW	Thall and Wathen

TABLE A2
Properties of various patient allocation procedures, where $p_0 = 0.25$ and $p_1 = 0.45$. Results are from 10^4 trial replicates.

		Patient Benefit Metrics		
n	**Procedure**	$N_1 - N_0$	$\hat{S}_{0.1}$	**ENS**
200	ER	0 (-28, 28)	0.069	70 (6.8)
	PBR	0	0	70 (6.6)
	Oracle	200	0	90 (7.0)
	TS	147 (-54, 194)	0.032	85 (9.5)
	FLGI($b = 5$)	165 (48, 194)	0.014	86 (8.6)
	FLGI($b = 10$)	164 (56, 192)	0.014	86 (8.6)
	TW(1/2)	124 (24, 182)	0.008	82 (8.2)
	TW($i/2n$)	88 (20, 148)	0.001	79 (7.6)
	RPW	30 (-2, 64)	0.000	73 (7.1)
	DBCD	30 (6, 58)	0.000	73 (6.6)
	ERADE	29 (8, 52)	0.000	73 (6.6)
	DTL	30 (10, 50)	0.000	73 (7.1)
